# Total Bile Acids: A Game-Changer in Predicting Short-Term Outcomes in AIS Patients Undergoing Thrombolysis

**DOI:** 10.7150/ijms.108310

**Published:** 2025-07-25

**Authors:** Zhuohang Liu, Min Chu, Zengyu Zhang, Jing Zhao

**Affiliations:** 1 Department of Neurology, Minhang Hospital, Fudan University, Shanghai, China.; 2 Institute of Healthy Yangtze River Delta, Shanghai Jiao Tong University, China.; 3 Department of Neurology, Zhongshan Hospital, Fudan University, Shanghai 200030, China.

**Keywords:** acute ischemic stroke, total bile acids, prognosis, endovascular therapy, intravenous thrombolysis

## Abstract

**Introduction:** Total bile acids (TBAs) are emerging as potential prognostic biomarkers in acute ischemic stroke (AIS). This study aimed to evaluate the association between TBA levels and long-term outcomes in AIS patients receiving intravenous thrombolysis (IVT).

**Methods:** A total of 231 AIS patients treated with IVT were prospectively enrolled. TBA levels were measured on admission. The primary outcome was the 3-month modified Rankin Scale (mRS) score. Logistic regression, restricted cubic splines (RCS), and decision curve analysis (DCA) were used to assess associations. Machine learning (ML) models were employed to validate predictive performance.

**Results:** High TBA (> 5 μmol/L) patients showed significantly better functional outcomes (91.2% vs 60.2%, P < 0.001). Multivariate analysis confirmed higher TBA independently predicted favorable outcomes (adjusted OR = 0.74, 95%CI:0.59-0.93), with TBA-integrated models showing superior discrimination (AUC = 0.970 vs ≤ 0.64 for NIHSS/TOAST). Restricted cubic spline analysis revealed a J-shaped non-linear relationship between TBA levels and outcome probability. Critically, a predictive model combining TBA with clinical factors demonstrated superior discriminative ability (AUC = 0.970), significantly outperforming traditional scores (NIHSS AUC = 0.64; TOAST AUC = 0.55). Decision curve analysis confirmed the model's clinical utility. Machine learning validation, particularly using Random Forest (accuracy: 93.8%, AUC: 93.14%, Brier score: 0.072), further substantiated TBA's predictive value. Feature importance analysis identified TBA (25.85) and hemoglobin (24.34) as the primary predictors, substantially exceeding others (e.g., NT-proBNP:3.60; admission NIHSS: 3.41; eGFR-EPI: 3.28).

**Conclusion:** TBA is independently associated with functional outcomes after IVT and may serve as a novel prognostic biomarker in AIS.

## Introduction

Acute ischemic stroke (AIS) is a serious medical emergency characterised by a sudden interruption of blood flow to the brain, resulting in ischemia and hypoxia in brain tissue. If not restored in time, it can trigger a series of severe neurological impairments 1, 2. Rapid and effective treatment is required to minimise brain damage and enhance recovery potential. Intravenous thrombolytic therapy (IVT), the standard treatment for AIS, has been shown to significantly improve patient prognosis 3. However, the efficacy of IVT is influenced by a variety of factors including “time window”, the underlying health status of patients, and complications after vascular recanalization. In recent years, studies have begun to focus on the products of the gut microbiome. These substances are not only involved in digestion but also influence stroke function and prognosis via the gut-brain axis 4-6. This could offer a new direction for predicting and intervening in the prognosis of stroke.

Bile acids are linked to lipid metabolism and have neuroprotective effects. They are also involved in neurological disorders, highlighting the significance of the gut-brain axis in recovery 7. For example, in traumatic brain injury (TBI), BA may be involved in regulating signalling pathways associated with apoptosis and oxidative stress, which in turn affect the recovery process after injury 8. In addition, BA may also play a role by affecting neuroinflammatory and immune responses 9. Studies suggest that changes in TBA levels in patients with AIS may reflect an imbalance in the gut microbiota that is closely linked to stroke pathogenesis and prognosis 10, 11. The literature indicates that elevated serum levels of total bile acids on admission are associated with reduced mortality within 3 months in patients with acute ischemic stroke 12, suggesting that TBA levels may be a predictor of the risk of death in the early post-stroke period. Another prospective follow-up study found that reduced bile acid excretion was an independent risk factor for stroke and death 11, highlighting the potential importance of maintaining appropriate bile acid levels in stroke prevention. However, no study to date has examined the relationship between serum TBA levels and poor functional prognosis in patients with IVT after AIS. Furthermore, the relationship between TBA levels and poor functional prognosis has not been explored based on stroke etiology.

In this study, we examined the relationship between baseline serum TBA levels and functional prognosis in AIS patients receiving intravenous thrombolysis. We also explored how this relationship may vary based on stroke etiology, using data collected from Minhang Hospital of Fudan University between 2018 and 2022.

## Method

### Study Design

This prognostic cohort study included patients with AIS who received IVT at Minhang Hospital of Fudan University between January 2018 and December 2022. Inclusion criteria were: (1) age ≥ 18 years with AIS confirmed by CT or MRI; (2) eligibility for IVT, defined as onset-to-treatment time ≤ 4.5 hours, CT-ASPECTS ≥ 6, normal coagulation profile, and well-controlled blood pressure; and (3) documented baseline NIH Stroke Scale (NIHSS) score. Exclusion criteria included severe hepatic or renal dysfunction (ALT/AST >3×ULN, eGFR < 30 mL/min/1.73 m^2^), active bleeding, malignancy, pre-existing disability (mRS ≥ 2), life expectancy < 3 months, or incomplete follow-up. The study protocol was approved by the Ethics Committee of Minhang Hospital, Fudan University.

Three-month functional outcomes were assessed through structured telephone interviews and in-person evaluations using the modified Rankin Scale (mRS). Favorable outcomes were defined as mRS ≤ 2, and unfavorable outcomes as mRS ≥ 3. After rigorous eligibility screening and data validation, a total of 231 patients were included in the final analysis.

### Data Collection

Fasting venous blood samples were collected within 24 hours of admission, prior to the administration of thrombolytic therapy. Serum total bile acid (TBA) levels were measured using enzymatic assays on the Roche Cobas 8000 analyzer (Roche Diagnostics, Indianapolis, IN, USA), serving as a key biomarker. Hematological parameters—including leukocyte differential counts (neutrophils, lymphocytes, monocytes), platelet count, and hemoglobin levels—were analyzed using the Sysmex XN-9000 automated hematology analyzer. Biochemical profiles encompassed lipid metabolism markers (total cholesterol, HDL-C, LDL-C, triglycerides), estimated glomerular filtration rate (eGFR) using the EPI formula, homocysteine, and N-terminal pro-brain natriuretic peptide (NT-proBNP). These were all measured on the Cobas 8000 platform using standardized enzymatic and immunoturbidimetric methods. To ensure sample integrity, all specimens were promptly centrifuged, aliquoted, and stored at -80 °C until batch analysis. Laboratory technicians were blinded to clinical outcomes to prevent measurement bias.

### Statistical Analysis

​All statistical analyses were conducted using R software (version 4.3; R Foundation for Statistical Computing, Vienna, Austria). Baseline characteristics were summarized and compared between groups using the tableone package. Categorical variables (e.g., sex, comorbidities) were analyzed using the chi-square test or Fisher's exact test, while continuous variables (reported as medians with interquartile ranges) were compared using the Mann-Whitney U test.

Associations between TBA levels and clinical outcomes were assessed through univariate and multivariate logistic regression analyses using the glm function. Variables with a p-value < 0.1 in the univariate analysis, along with age and sex, were included in the multivariate model 13. To assess multicollinearity among variables in the multivariate regression model, the Variance Inflation Factor (VIF) was calculated using the car package. All variables exhibited VIF values below 5, indicating no significant multicollinearity.

The predictive utility of TBA for clinical outcomes following IVT in AIS patients was evaluated using receiver operating characteristic (ROC) curve analysis to quantify discriminative capacity. This was complemented by decision curve analysis (DCA) for clinical net benefit, and assessments of calibration (Brier score), reclassification [Net Reclassification Improvement (NRI)], and discrimination [Integrated Discrimination Improvement (IDI)]. Calibration of the predictive model was assessed using the calibrate function from the rms package, employing the bootstrap method with 1,000 resamples.

For machine learning model development, statistically significant variables from multivariate analyses were incorporated into eight algorithms: XGBoost, decision tree, logistic regression, multilayer perceptron (MLP), naive Bayes, k-nearest neighbors (k-NN), random forest, and support vector machine (SVM), implemented via the tidymodels package. Automated hyperparameter tuning was applied to optimize model performance, with 1,000 bootstrap resamples generated using the 'bootstraps ()' function to rigorously evaluate model stability and generalization. Model performance was assessed using the area under the ROC curve (AUC) for classification accuracy, prediction accuracy for overall correctness, and the Brier score (range: 0-1; lower values indicating better calibration) to evaluate probabilistic prediction fidelity. The bootstrap approach provided robust estimates of model variability and reduced overfitting risks compared to single validation splits.

## Results

### Clinical Characteristics of Patients

**Figure 1:**
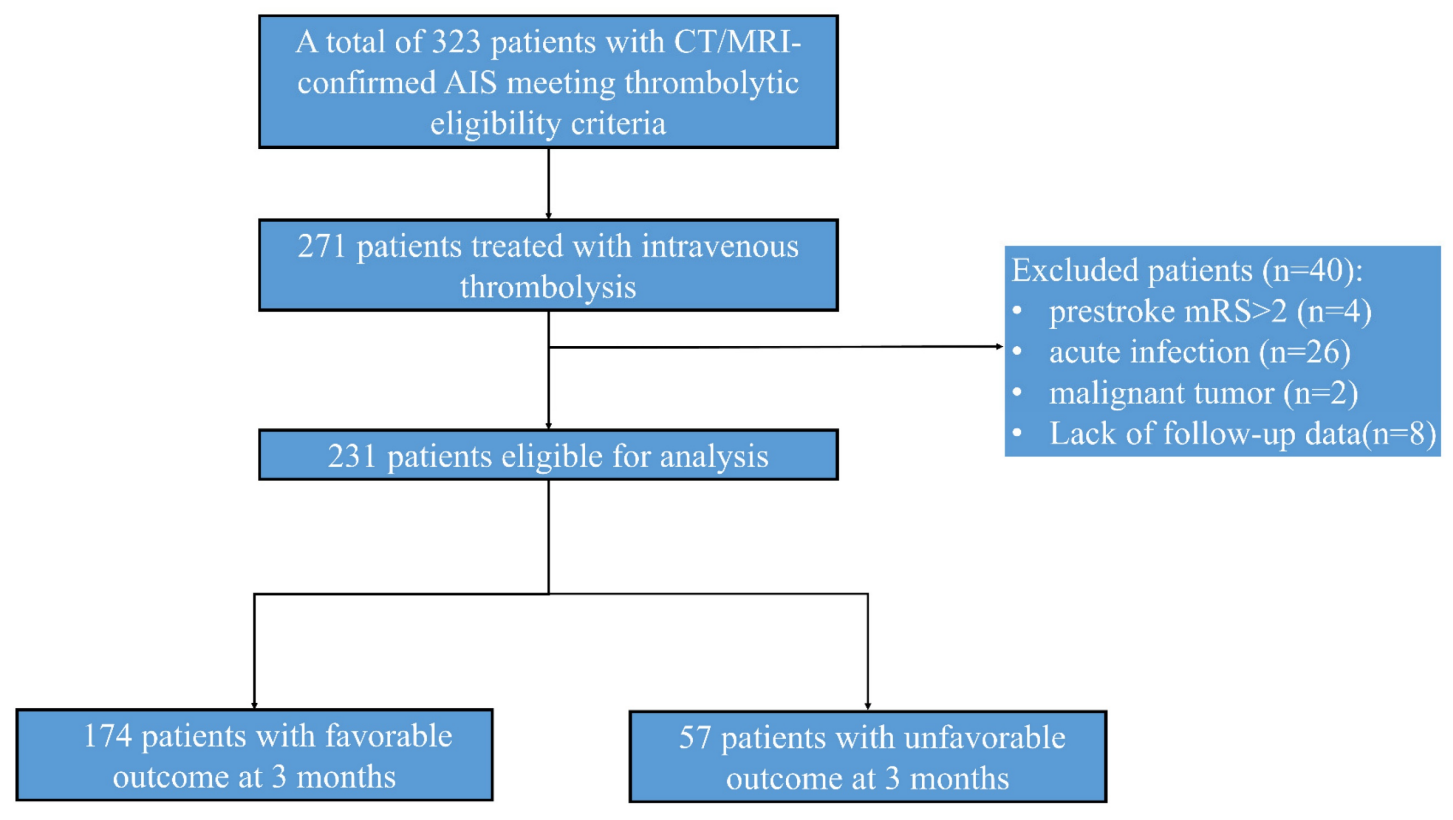
** Diagram of the study recruitment**.

A consecutive cohort of 410 AIS patients receiving endovascular therapy was initially enrolled. After excluding patients with prestroke mRS score ≥ 2 (n = 4), active infections (n = 26; pneumonia = 18, cholecystitis = 2), malignancy (n = 2), and lost follow-up (n=8), 231 participants were included in final analyses (Fig. 1).

The cohort comprised 65.5% males in Group A versus 71.9% in Group B (P = 0.371), with mean ages of 68.5±8.2 and 71.4±9.1 years respectively (P = 0.099). Both groups demonstrated comparable prevalence of modifiable risk factors including heavy alcohol consumption (32.1% vs 35.6%), smoking (44.3% vs 47.2%), hypertension (68.9% vs 71.4%), and diabetes mellitus (39.2% vs 42.1%). Neurological severity differed significantly between groups, with median admission NIHSS scores of 4 (IQR: 2-6) versus 6 (IQR: 4-8), improving to 1 (IQR: 0-2) versus 5 (IQR: 3-7) at discharge (P < 0.001).

**Figure 2:**
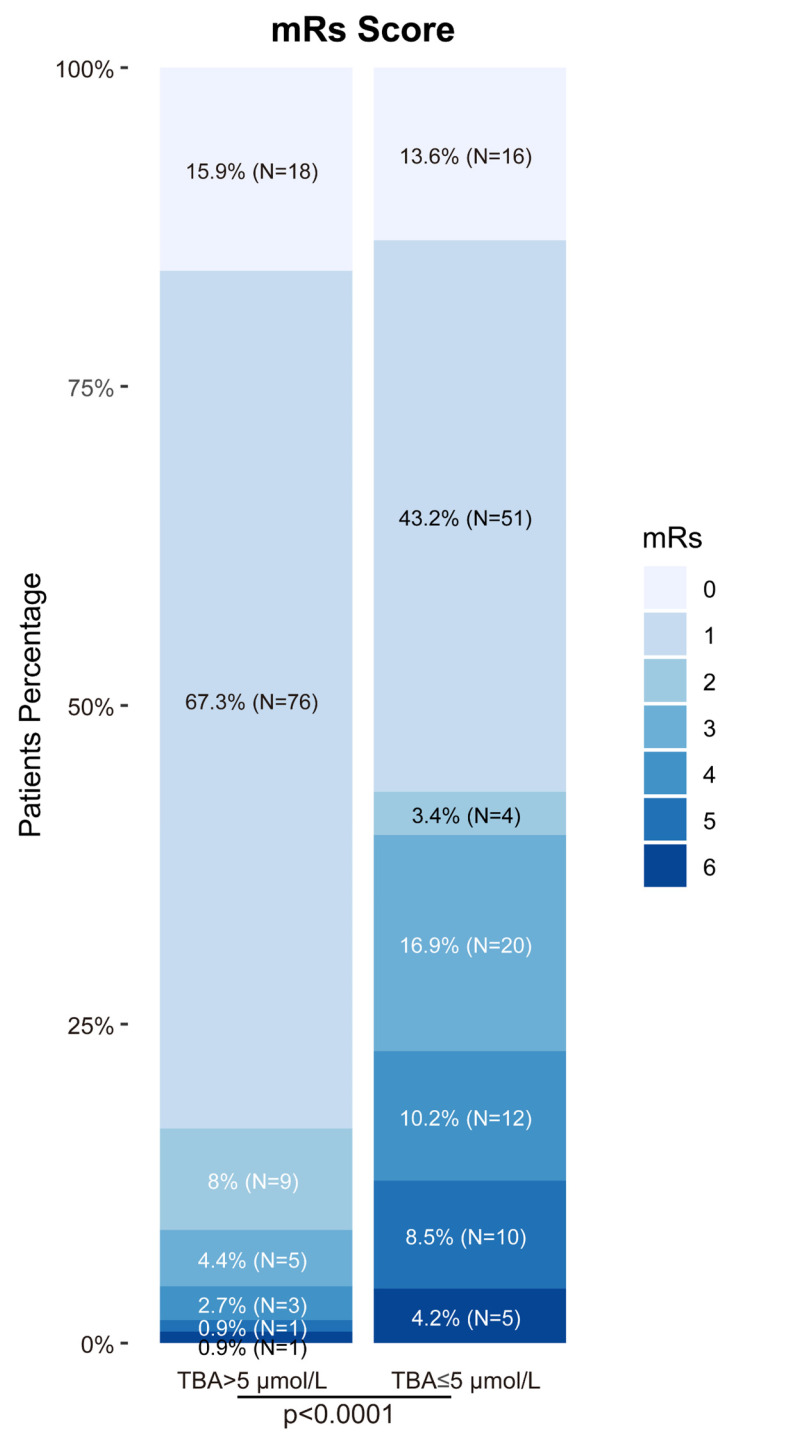
** Distribution of mRs score at 90 days among patients after thrombolysis.** The proportion of patients with high versus low TBA levels across different prognostic neurological function scores.TBA threshold=5 μmol/L.

Biomarker analysis revealed significant intergroup differences in triglycerides (1.8 vs 2.4 mmol/L, P = 0.012), lymphocyte counts (1.1 vs 0.8×10⁹/L, P = 0.003), and platelet counts (218 vs 245×10⁹/L, P = 0.021). Notably, TBA levels showed strong prognostic correlation: patients with favorable outcomes exhibited significantly higher median TBA levels [5.6 μmol/L (IQR:4.5-8.5), P < 0.001] compared to those with poorer outcomes [2.4 μmol/L (IQR:1.2-3.8), Table 1]. This association was reinforced by functional outcomes, with 91.2% of TBA > 5 μmol/L patients achieving admission mRS 0-2 versus 60.2% in TBA ≤ 5 μmol/L group (Fig. 2). Other biochemical parameters demonstrated inverse correlations with positive outcomes (Table 1).

### Uni- and Multi-Variate Analysis of Factors Related to Unfavorable Outcome TBA

**Figure 3:**
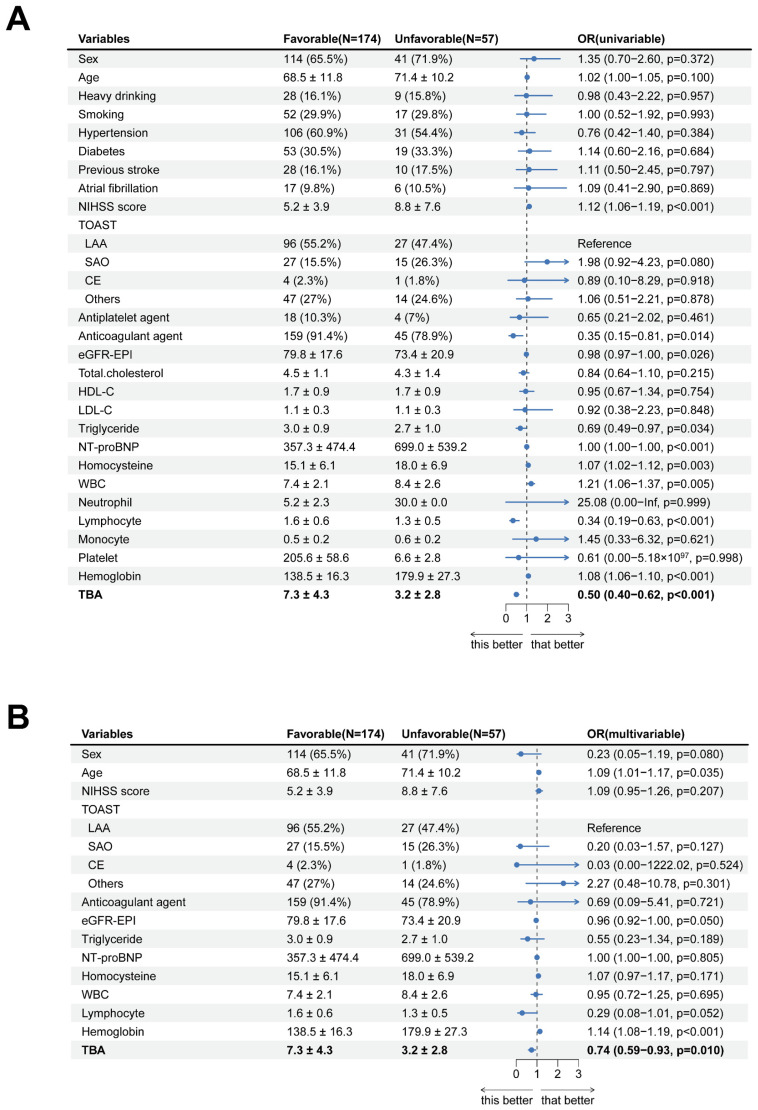
** Forest diagram of logistics regression analysis described AIS patients 3-month outcome. (A, B)** Uni- and Multi-variate regression analysis of characteristics.

In a logistic regression study of 231 acute ischemic stroke patients, we investigated the association between clinical characteristics and 3-month functional outcomes. Univariate analysis demonstrated significant associations for age (P = 0.100) and admission NIHSS scores (P < 0.001) with prognosis. However, multivariate modeling revealed divergent findings: while anticoagulant therapy (P = 0.014) and hemoglobin levels (P < 0.001) emerged as independent predictors, the prognostic significance of NIHSS scores was not retained (P = 0.207). Notably, elevated TBA levels were significantly linked to favorable outcomes in univariate analysis (OR = 0.50, 95% CI 0.40-0.62; P < 0.001). This relationship persisted in multivariate analysis (OR = 0.74, 95% CI 0.59-0.93; P = 0.010), though the effect size attenuation suggested potential interaction with other prognostic determinants (Table 2, Fig. 3).

**Figure 4:**
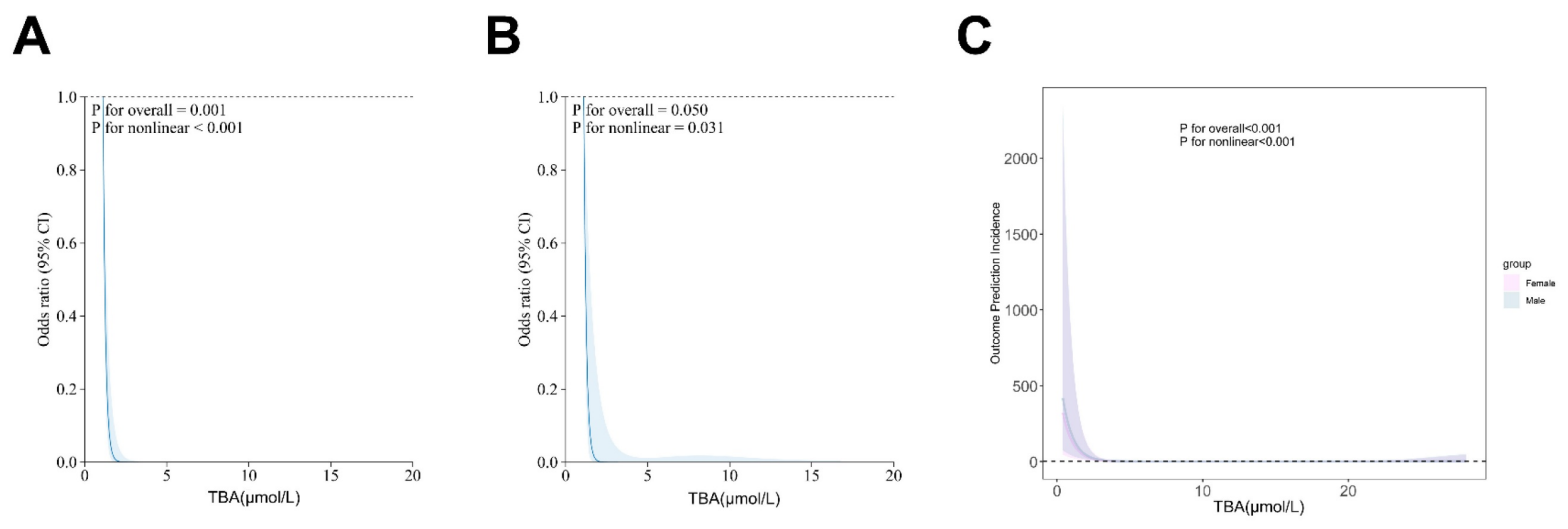
** Restricted cubic spline for associations between TBA and clinical outcomes in AIS patients**.** (A, B)** The relation of TBA with the odds ratios from the logistic regression model after or without adjustment for the variables.** (C)** The association of TBA with the odds ratios by sex group.

**Figure 5:**
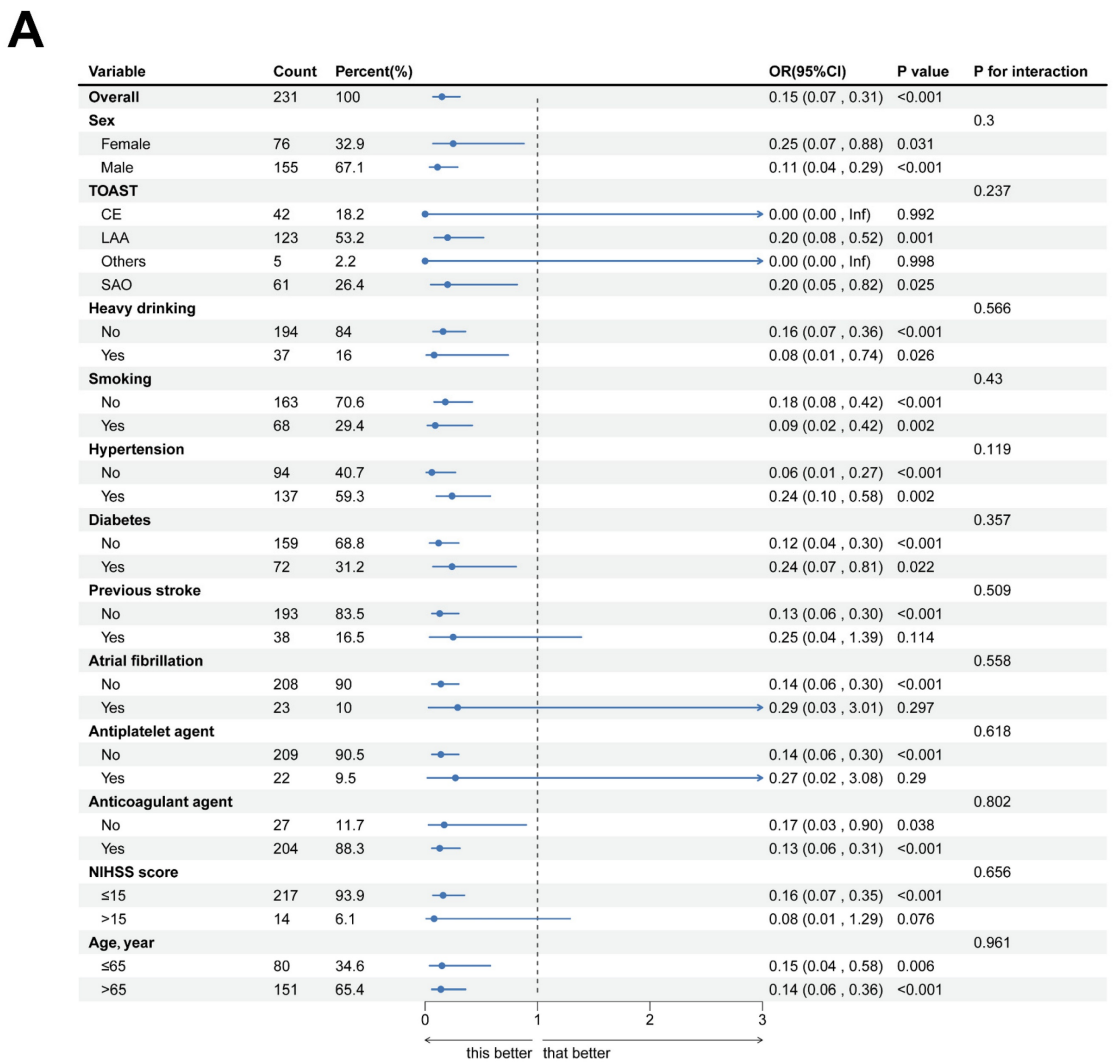
** Subgroup analyses of the primary outcome. (A)** Shown is associations of total bile acids with functional outcome in the subgroup.

Our analytical approach was conducted in two phases. First, we performed univariate screening with a p-value threshold of < 0.1. Then, we applied multivariate adjustment, taking into account established confounders. Restricted cubic spline (RCS) analysis revealed a significant J-shaped relationship between TBA levels and stroke outcomes (overall P = 0.0119, nonlinear P < 0.001), a finding that remained consistent in sex-stratified models (Fig. 4). Subgroup analyses further confirmed the prognostic value of TBA, showing similar effects across different groups, including those based on antiplatelet use, atrial fibrillation status, stroke history, baseline NIHSS, and TOAST classification. Notably, patients with TBA levels greater than 5 μmol/L had a significantly lower risk compared to those with TBA levels ≤5 μmol/L (Fig. 5). These findings highlight the complex role of TBA in stroke prognosis.

### Association of TBA with Clinical Outcome

**Figure 6:**
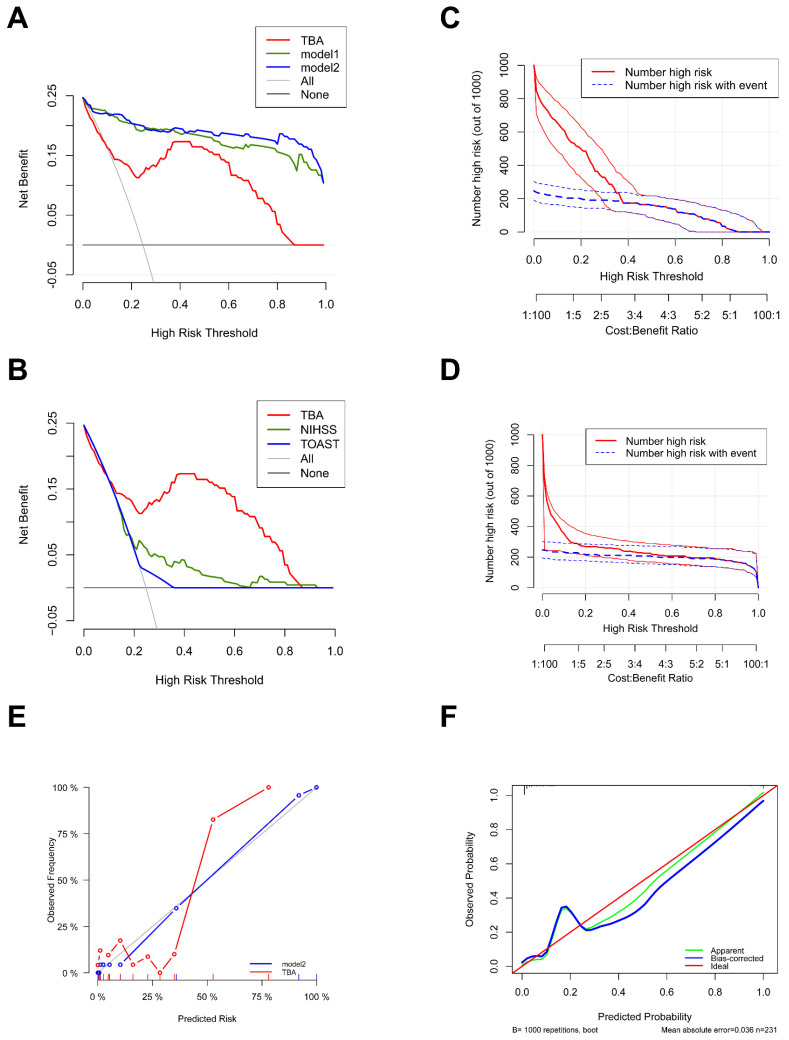
**Calibration plot and decision curve analysis (DCA) of TBA. (A, B)** DCA plots assessing the diagnostic utility of TBA, Model 1, and Model 2 (a), and NIHSS and TOAST (c) across different threshold probabilities. The y-axis represents net benefit values ranging from 0.05 to 1.0. Strategies include a threshold-based approach (TBA), intervention for all patients ("All"), and no intervention ("None"). **(C, D)** Clinical impact curves (CIC) describing the diagnostic efficacy of TBA with or without adjustment (Model 2). **(E)** Calibration curve showing the relationship between predicted and actual observations for TBA, with or without adjustment (Model 2). **(F)** Calibration curve comparing predicted (x-axis) and observed probabilities (y-axis) using bootstrap sampling (1000 repetitions) for TBA with adjustment. Model 1: Sex, Age, NIHSS score at admission, TOAST, Anticoagulant agent, eGFR-EPI, Triglycerides, NT-proBNP, Homocysteine, WBC, Lymphocyte, Hemoglobin. Model 2: Model 1+TBA.

Decision curve analysis revealed the superior clinical diagnostic utility of TBA across a comprehensive range of risk thresholds. Specifically, within the clinically relevant threshold range of 0.2-0.8, the net benefit of intervention guided by TBA alone were consistently exceeded by both model 1and model2. Comparative analysis demonstrated distinct performance patterns among models: At lower thresholds (0.0-0.3), TBA and NIHSS provided higher net benefits compared to TOAST, whereas TBA maintained its advantage at higher thresholds (0.6-0.8) (Figs. 6A, B).

Clinical impact curves further validated these findings, showing strong concordance between model-identified high-risk populations and actual cases at thresholds > 0.2. Notably, TBA predictions achieved nearly perfect alignment with true patient outcomes after covariate adjustment (Figs. 6C, D). Calibration analysis reinforced the models' reliability, with TBA, model1, and model2 all exhibiting close agreement between predicted and observed probabilities. The calibration curve for TBA showed minimal deviation from the ideal line (MAE = 0.036), while bootstrap validation with 1000 repetitions ensured robust error estimation (Figs. 6E, F). Therefore, the predicted probabilities are broadly in good accordance with the actual probabilities.

### Machine Learning Validation of TBA-Integrated Models

**Figure 7:**
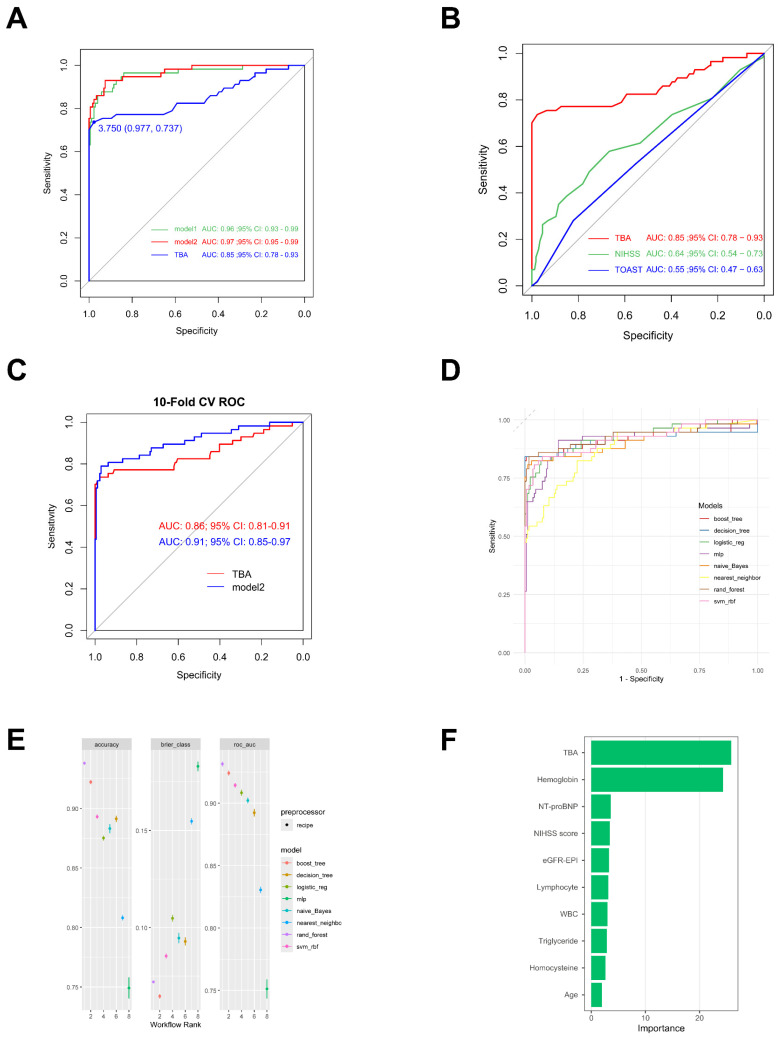
** Receiver Operating Characteristic (ROC) curve of TBA. (A, B)** ROC illustrating the diagnostic performance of TBA alone, model1, model2, NIHSS and TOAST with the area under the curve (AUC) indicated.** (C)** 10-Fold Cross-Validation ROC Curves for TBA and Model2. **(D, E)** ROC demonstrateing diagnostic efficacy of various ML methods.** (F)** Variable Importance Plot from Random Forest Model. boost_tree: Boosted Tree; decision_tree: Decision Tree; logistic_reg: Logistic Regression; naive_Bayes: Naive Bayes; nearest_neighbor: k-Nearest Neighbors; rand_forest: Random Forest; svm_rbf: Support Vector Machine with RBF Kernel.

The AUC serves as a key metric for evaluating model predictive performance, where higher values denote superior predictive capacity. TBA demonstrates robust predictive ability with an AUC of 0.85. Notably, its integration with other clinical parameters in Model 1 (baseline AUC: 0.96) significantly enhanced prognostic performance, achieving an AUC of 0.97 (Model2,95% CI: 0.95-0.99; Fig. 7A). Comparative analysis revealed TBA's superior discriminative power (AUC: 0.85, 95% CI: 0.78-0.93) relative to NIHSS (AUC: 0.64, 95% CI: 0.54-0.73) and TOAST (AUC: 0.55, 95% CI: 0.47-0.63; Fig. 7B). Ten-fold cross-validated ROC analysis showed Model 2 (AUC: 0.91, 95% CI: 0.85-0.97) outperforming the TBA-only model (AUC: 0.86, 95% CI: 0.81-0.91; Fig. 7C). Although the C-statistic improvement following TBA incorporation was non-significant (p = 0.321), the composite model demonstrated clinically meaningful enhancements in risk stratification. This was evidenced by significant continuous net reclassification improvement (NRI = 0.405, 95% CI: 0.137-0.673; p = 0.003) and integrated discrimination improvement (IDI = 0.033, 95% CI: 0.010-0.056; p = 0.004; Table 3).

Multimodel validation across eight machine learning architectures confirmed the prognostic robustness of TBA-integrated models. The random forest implementation achieved optimal performance metrics: accuracy = 93.80%, AUC = 93.14%, and Brier score = 0.072 (Figs. 7D, E). Feature importance analysis identified TBA (25.85) and hemoglobin (24.34) as primary predictors, substantially outperforming other variables including NT-proBNP (3.60), admission NIHSS (3.41), and eGFR-EPI (3.28; Fig. 7F).

These findings collectively demonstrate that TBA-enhanced models provide statistically significant improvements in risk discrimination accuracy and clinical utility for prognostic stratification.

## Discussion

In this study, we investigated the correlation between TBA levels and the 3-month functional outcome in acute ischemic stroke (AIS) patients treated solely with intravenous thrombolysis (IVT). Our findings revealed that AIS patients who exhibited favorable 3-month outcomes post-IVT had elevated TBA levels compared to those with unfavorable outcomes. Additionally, we established TBA as an independent predictor of prognostic outcomes. Importantly, when combined with traditional risk factors, TBA exhibited enhanced prognostic predictive value. These results underscore the significance of TBA in assessing prognostic outcomes in AIS patients and provide valuable insights for individualized treatment and management strategies.

The relationship between gut flora and stroke prognosis is an emerging area of research and its potential clinical significance is gradually being recognized 14. Gut flora is a complex microbial ecosystem in the human body, and they are involved in various physiological processes, including metabolism, immune regulation, and host-neural communication 15-17. After stroke onset, the composition and metabolic activity of the gut flora may change, and these changes may influence the stroke recovery process through the so-called gut-brain axis 18. Furthermore, an alteration in the composition of the intestinal flora may result in impaired intestinal barrier function, increasing the entry of inflammatory mediators of intestinal origin into the circulation, which in turn affects the prognosis of stroke 19, 20. Studies suggest that gut flora-derived metabolites, such as short-chain fatty acids (SCFAs), may exert modulatory effects on the immune response and neural repair after stroke 21, 22. SCFAs, including acetate, propionate, and butyrate, have been shown to reduce neuroinflammation, promote neuronal cell survival, and synaptic plasticity, thereby potentially improving neurological recovery after stroke 23. Thus, gut microbiota-derived metabolites might modulate the development and outcome of stroke.

Bile acids, once considered solely digestive agents, are now recognized as systemic signaling molecules shaped by host-microbiome interactions 24. They influence a range of physiological processes, including inflammation and metabolism, through the gut-brain axis. Disruption of bile acid homeostasis has been linked to gut microbial dysbiosis, a condition increasingly associated with stroke risk and recovery 25. Ischemic stroke induces an imbalance in gut microbiota, leading to a reduction in microbiota-mediated bile acids, particularly ursodeoxycholic acid (UDCA) 26, 27. Restoring UDCA alleviates stroke-induced pathological damage by reducing infarction size and improving neurological and cognitive function, likely through the TGR5/PKA pathway, highlighting UDCA as a potential therapeutic target for ischemic stroke 28. In a cohort of patients with acute ischemic stroke (AIS) treated with thrombolysis, we found that higher serum total bile acid (TBA) concentrations were consistently associated with better functional outcomes 12. This association, confirmed through restricted cubic spline modeling and machine learning algorithms, is consistent with prior studies that identify impaired bile acid metabolism as an independent risk factor for cerebrovascular events.

Bile acids undergo microbial transformation in the gut, generating secondary metabolites that engage nuclear (FXR) and membrane-bound (TGR5) receptors 29, 30. However, stroke-induced disruption of the gut barrier can alter the microbial composition, potentially reducing the production of neuroprotective bile acid derivatives. In turn, impaired bile acid signaling may worsen neurovascular injury, establishing a bidirectional feedback loop between brain ischemia and gut dysfunction 31. Post-stroke alterations in microbiota composition and bile acid signaling have been linked to poorer neurological outcomes, as supported by experimental models 32. Notably, activation of TGR5 on microglia attenuates inflammatory responses, while FXR modulates astrocyte and endothelial cell function 28, 33. These pathways suggest that bile acids may mediate stroke recovery via immunometabolic mechanisms. Our findings suggest three promising therapeutic strategies. These include modulating the gut microbiome, targeting bile acid receptors pharmacologically, and using bile acid profiles as dynamic biomarkers for monitoring. Future studies should test whether these associations are causal and define the dose-response relationships between specific bile acid species and clinical outcomes.

Although the present study provides strong evidence for TBA as a prognostic biomarker in patients after thrombolysis for AIS, we recognise the limitations of the study. First, the sample size of this study may limit the generalised ability of the results. Second, the study design was a prospective analysis, which may be subject to selection bias and information bias. In addition, we were unable to explore in detail the interactions between TBA and gut microbiota, an important aspect to focus on in future studies.

## Conclusion

In summary, this study reveals the potential clinical value of TBA in patients with AIS, especially in assessing prognosis after IVT treatment. Although there are some limitations, our study provides a new direction for future research and offers clinicians new tools for treatment and prognosis assessment. With further understanding of the mechanism of action of TBA in brain diseases, we expect to be able to develop new therapeutic strategies to improve clinical outcomes in patients with AIS.

## Figures and Tables

**Table 1:** Baseline characteristics of AIS stroke patients receiving IVT therapy based on favorable vs. unfavorable outcome at 3 months

Characteristics	Overall (n=231)	Favorable (n=178)	Unfavorable (n=57)	p
Demographics				
Sex = Male (n, %)	155 (67.1)	114 (65.5)	41 (71.9)	0.464
Age (median [IQR], year)	69.0 [63.5, 77.0]	68.0 [63.0, 76.8]	70.0 [65.0, 78.0]	0.125
Medical history				
Heavy drinking = Yes (n, %)	37 (16.0)	28 (16.1)	9 (15.8)	1.000
Smoking = Yes (n, %)	69 (29.9)	52 (29.9)	17 (29.8)	1.000
Hypertension = Yes (n, %)	137 (59.3)	106 (60.9)	31 (54.4)	0.474
Diabetes = Yes (n, %)	72 (31.2)	53 (30.5)	19 (33.3)	0.809
Previous stroke = Yes (n, %)	38 (16.5)	28 (16.1)	10 (17.5)	0.959
Atrial fibrillation = Yes (n, %)	23 (10.0)	17 (9.8)	6 (10.5)	1.000
Antiplatelet agent = Yes (n, %)	22 (9.5)	18 (10.3)	4 (7.0)	0.606
Anticoagulant agent = Yes (n, %)	204 (88.3)	159 (91.4)	45 (78.9)	**0.022**
NIHSS score (median [IQR])	5.0 [3.0, 8.0]	4.0 [3.0, 6.0]	6.0 [3.0, 13.0]	**0.002**
TOAST (n, %)				0.337
LAA	123 (53.2)	96 (55.2)	27 (47.4)	
SAO	61 (26.4)	47 (27.0)	14 (24.6)	
CE	42 (18.2)	27 (15.5)	15 (26.3)	
Others	5 (2.2)	4 (2.3)	1 (1.8)	
Laboratory measures				
eGFR-EPI (median [IQR], mL/min/1.73m²)	83.1 [69.8, 89.4]	84.4 [72.5, 89.9]	75.5 [65.6, 87.9]	**0.03**
Total cholesterol (median [IQR], mmol/L)	4.3 [3.7, 5.0]	4.4 [3.8, 5.1]	4.1 [3.5, 4.8]	0.069
HDL-C (median [IQR], mmol/L)	1.4 [1.0, 2.1]	1.5 [1.1, 2.1]	1.4 [1.0, 2.0]	0.38
LDL-C (median [IQR], mmol/L)	1.1 [0.9, 1.3]	1.1 [0.9, 1.3]	1.1 [0.9, 1.4]	0.914
Triglyceride (median [IQR], mmol/L)	2.8 [2.2, 3.4]	2.9 [2.4, 3.5]	2.6 [1.9, 3.3]	**0.023**
NT-proBNP (median [IQR], pg/mL)	214.0 [83.0, 694.9]	152.0 [71.2, 383.8]	724.9 [258.0, 1261.3]	**< 0.001**
Homocysteine (median [IQR], μmol/L)	14.0 [11.2, 18.7]	13.2 [10.9, 17.4]	16.1 [13.2, 22.9]	**0.005**
WBC (median [IQR], ×10⁹/L)	7.2 [6.0, 9.1]	7.0 [5.9, 8.7]	8.2 [6.6, 10.3]	**0.004**
Neutrophil (median [IQR], ×10⁹/L)	5.4 [4.1, 15.1]	4.6 [3.7, 6.1]	132.0 [120.0, 144.0]	**< 0.001**
Lymphocyte (median [IQR], ×10⁹/L)	1.5 [1.1, 1.9]	1.6 [1.2, 2.0]	1.2 [0.9, 1.7]	**0.001**
Monocyte (median [IQR], ×10⁹/L)	0.5 [0.4, 0.7]	0.5 [0.4, 0.7]	0.6 [0.4, 0.7]	0.798
Platelet (median [IQR], ×10⁹/L)	178.0 [101.0, 228.0]	204.5 [163.2, 246.0]	6.1 [4.4, 8.8]	**< 0.001**
Hemoglobin (median [IQR], g/dL)	142.0 [132.0, 161.5]	137.0 [129.0, 147.0]	187.0 [159.0, 217.0]	**< 0.001**
TBA (median [IQR], μmol/L)	5.0 [3.9, 7.4]	5.6 [4.5, 8.5]	2.4 [1.2, 3.8]	**< 0.001**

Continuous variables are expressed as the median (interquartile range). Categorical variables are expressed as the frequency (percentage). Abbreviations: eGFR-EPI: Glomerular.filtration.rate_eGFR.EPI; NT-proBNP: N-terminal pro-brain natriuretic peptide; LDL-C: low-density lipoprotein cholesterol; HDL-C: high-density lipoprotein cholesterol; WBC White Blood Cell;NIHSS: National Institutes of Health Stroke Scale.

**Table 2:** Univariate and Multivariate regression analysis of factors related to 3-month outcome

Variables		Favorable (N=174)	Unfavorable (N=57)	OR (univariable)	OR (multivariable)
Sex	Male	114 (65.5%)	41 (71.9%)	1.35 (0.70-2.60, p=.372)	0.23 (0.05-1.19, p=.080)
Age, years	Mean ± SD	68.5 ± 11.8	71.4 ± 10.2	1.02 (1.00-1.05, p=.100)	**1.09 (1.01-1.17, p=.035)**
Heavy drinking	Yes	28 (16.1%)	9 (15.8%)	0.98 (0.43-2.22, p=.957)	
Smoking	Yes	52 (29.9%)	17 (29.8%)	1.00 (0.52-1.92, p=.993)	
Hypertension	Yes	106 (60.9%)	31 (54.4%)	0.76 (0.42-1.40, p=.384)	
Diabetes	Yes	53 (30.5%)	19 (33.3%)	1.14 (0.60-2.16, p=.684)	
Previous stroke	Yes	28 (16.1%)	10 (17.5%)	1.11 (0.50-2.45, p=.797)	
Atrial fibrillation	Yes	17 (9.8%)	6 (10.5%)	1.09 (0.41-2.90, p=.869)	
NIHSS score	Mean ± SD	5.2 ± 3.9	8.8 ± 7.6	1.12 (1.06-1.19, p<.001)	1.09 (0.95-1.26, p=.207)
TOAST	LAA	96 (55.2%)	27 (47.4%)		
	SAO	27 (15.5%)	15 (26.3%)	1.98 (0.92-4.23, p=.080)	0.20 (0.03-1.57, p=.127)
	CE	4 (2.3%)	1 (1.8%)	0.89 (0.10-8.29, p=.918)	0.03 (0.00-1222.02, p=.524)
	Others	47 (27%)	14 (24.6%)	1.06 (0.51-2.21, p=.878)	2.27 (0.48-10.78, p=.301)
Antiplatelet agent	Yes	18 (10.3%)	4 (7%)	0.65 (0.21-2.02, p=.461)	
Anticoagulant agent	Yes	159 (91.4%)	45 (78.9%)	0.35 (0.15-0.81, p=.014)	0.69 (0.09-5.41, p=.721)
eGFR-EPI, mL/min/1.73m²	Mean ± SD	79.8 ± 17.6	73.4 ± 20.9	0.98 (0.97-1.00, p=.026)	0.96 (0.92-1.00, p=.050)
Total cholesterol, mmol/L	Mean ± SD	4.5 ± 1.1	4.3 ± 1.4	0.84 (0.64-1.10, p=.215)	
HDL-C, mmol/L	Mean ± SD	1.7 ± 0.9	1.7 ± 0.9	0.95 (0.67-1.34, p=.754)	
LDL-C, mmol/L	Mean ± SD	1.1 ± 0.3	1.1 ± 0.3	0.92 (0.38-2.23, p=.848)	
Triglyceride, mmol/L	Mean ± SD	3.0 ± 0.9	2.7 ± 1.0	0.69 (0.49-0.97, p=.034)	0.55 (0.23-1.34, p=.189)
NT-proBNP, pg/mL	Mean ± SD	357.3 ± 474.4	699.0 ± 539.2	1.00 (1.00-1.00, p<.001)	1.00 (1.00-1.00, p=.805)
Homocysteine, μmol/L	Mean ± SD	15.1 ± 6.1	18.0 ± 6.9	1.07 (1.02-1.12, p=.003)	1.07 (0.97-1.17, p=.171)
WBC, ×10⁹/L	Mean ± SD	7.4 ± 2.1	8.4 ± 2.6	1.21 (1.06-1.37, p=.005)	0.95 (0.72-1.25, p=.695)
Neutrophil, ×10⁹/L	Mean ± SD	5.2 ± 2.3	30.0 ± 0.0	25.08 (0.00-Inf, p=.999)	
Lymphocyte, ×10⁹/L	Mean ± SD	1.6 ± 0.6	1.3 ± 0.5	0.34 (0.19-0.63, p<.001)	0.29 (0.08-1.01, p=.052)
Monocyte, ×10⁹/L	Mean ± SD	0.5 ± 0.2	0.6 ± 0.2	1.45 (0.33-6.32, p=.621)	
Platelet, ×10⁹/L	Mean ± SD	205.6 ± 58.6	6.6 ± 2.8	0.61 (0.00-5.18×10^97^, p=.998)	
Hemoglobin, g/dL)	Mean ± SD	138.5 ± 16.3	179.9 ± 27.3	1.08 (1.06-1.10, p<.001)	**1.14 (1.08-1.19, p<.001)**
TBA, μmol/L	Mean ± SD	7.3 ± 4.3	3.2 ± 2.8	0.50 (0.40-0.62, p<.001)	**0.74 (0.59-0.93, p=.010)**

**Table 3:** Reclassification and discrimination statistics for 3-month clinical outcome by TBA among patients with thrombolysis

	C statistics		NRI(Categorical)		NRI(Continuous)		IDI	
Model	Estimate (95% CI)	P value	Estimate [95% CI]	P value	Estimate [95% CI]	P value	Estimate [95% CI]	P value
model1	0.964 (0.934 - 0.994)		Reference		Reference		Reference	
Unfavorable outcome (mRS score 3-6)								
model1+ TBA (model2)	0.97 (0.946 - 0.995)	0.321	0.047 (-0.004 to 0.097)	0.069	0.405(0.137 to0.673)	0.003	0.033 (0.010 to 0.056)	0.004
